# Perceptions of Factors Influencing Engagement With Health and Well-being Apps in the United Kingdom: Qualitative Interview Study

**DOI:** 10.2196/29098

**Published:** 2021-12-16

**Authors:** Dorothy Szinay, Olga Perski, Andy Jones, Tim Chadborn, Jamie Brown, Felix Naughton

**Affiliations:** 1 School of Health Sciences University of East Anglia Norwich United Kingdom; 2 Department of Behavioural Science and Health University College London London United Kingdom; 3 Norwich Medical School University of East Anglia Norwich United Kingdom; 4 Behavioural Insights Public Health England London United Kingdom; 5 SPECTRUM Consortium London United Kingdom

**Keywords:** behavior change, health apps, mHealth, smartphone app, framework analysis, COM-B, TDF, user engagement, motivation, usability, engagement, mobile phone

## Abstract

**Background:**

Digital health devices, such as health and well-being smartphone apps, could offer an accessible and cost-effective way to deliver health and well-being interventions. A key component of the effectiveness of health and well-being apps is user engagement. However, engagement with health and well-being apps is typically poor. Previous studies have identified a list of factors that could influence engagement; however, most of these studies were conducted on a particular population or for an app targeting a particular behavior. An understanding of the factors that influence engagement with a wide range of health and well-being apps can inform the design and the development of more engaging apps in general.

**Objective:**

The aim of this study is to explore user experiences of and reasons for engaging and not engaging with a wide range of health and well-being apps.

**Methods:**

A sample of adults in the United Kingdom (N=17) interested in using a health or well-being app participated in a semistructured interview to explore experiences of engaging and not engaging with these apps. Participants were recruited via social media platforms. Data were analyzed with the framework approach, informed by the Capability, Opportunity, Motivation–Behaviour (COM-B) model and the Theoretical Domains Framework, which are 2 widely used frameworks that incorporate a comprehensive set of behavioral influences.

**Results:**

Factors that influence the capability of participants included available user guidance, statistical and health information, reduced cognitive load, well-designed reminders, self-monitoring features, features that help establish a routine, features that offer a safety net, and stepping-stone app characteristics. Tailoring, peer support, and embedded professional support were identified as important factors that enhance user opportunities for engagement with health and well-being apps. Feedback, rewards, encouragement, goal setting, action planning, self-confidence, and commitment were judged to be the motivation factors that affect engagement with health and well-being apps.

**Conclusions:**

Multiple factors were identified across all components of the COM-B model that may be valuable for the development of more engaging health and well-being apps. Engagement appears to be influenced primarily by features that provide user guidance, promote minimal cognitive load, support self-monitoring (capability), provide embedded social support (opportunity), and provide goal setting with action planning (motivation). This research provides recommendations for policy makers, industry, health care providers, and app developers for increasing effective engagement.

## Introduction

### Background

Smoking, physical inactivity, inadequate diet, and excessive alcohol consumption are the main risk factors for noncommunicable diseases that are responsible for >56.9 million deaths worldwide [1]. People with mental health problems often have poor physical health and vice versa [2,3]. A range of interventions has been developed to reduce the burden of ill health. The integration of multimedia technologies within the health care domain has led to the development of interventions that are delivered digitally via mobile phones, wearable devices, and smartphone apps. Smartphone apps are constantly available to the user and therefore act as portable tools for the delivery of easily accessible health and well-being interventions [4]. There is early evidence of the effectiveness of apps for physical inactivity [5-8], weight loss [7,9,10], alcohol reduction in nondependent drinkers [11], and mental health promotion [12]. Health apps are also considered a cost-effective solution [7,13] and have the potential to increase access for hard-to-reach populations that are resistant or unable to seek face-to-face support, for instance, because of stigma or geographical barriers [14].

Engagement is a necessary component for the effectiveness of a health or well-being app. Engagement with health and well-being apps can be defined as “(1) the extent (e.g. amount, frequency, duration, depth) of usage and (2) a subjective experience characterised by attention, interest and affect” [15]. However, it has been argued that measuring *effective engagement* is more important than simply the time spent on an app and the frequency of use [16]. Yardley et al [16] define *effective engagement* with a smartphone app as involving 2 components: the first is the intensity of engagement that is necessary for achieving desired outcomes, with sustained app engagement over a period of weeks, months, or even years (referred to as *microengagement*). However, microengagement alone is not sufficient for behavior change [16]. The Yardley et al [16] model also emphasizes engagement with the broader behavior change process and goals (ie, *macroengagement*), which is considered separate from, although intimately linked with, microengagement. On the basis of this distinction of microengagement and macroengagement with health and well-being apps, some factors may relate more to the former or the latter, with microengagement influencing macroengagement and vice versa. For example, engagement may be affected by common contextual factors, such as personal (eg, their interest), environmental (eg, where the engagement occurs and the individual’s lifestyle), or social context (eg, family or culture). Owing to the complexity of engagement, researchers recognize that it is difficult to define what constitutes *good* or *sufficient* engagement. Some individuals may require a longer period of engagement with an app than others for the desired behavior change to occur.

Despite the promise of health apps, engagement tends to be poor [17,18]. For example, a mobile consumer report found that for medical, health, and fitness apps, only 20% of users use the app 1 day after installation and only 8% after 7 days of installation [19]. A panel-based analysis systematically examined use patterns in 93 mental health apps and found that the median app retention rates at 15 and 30 days of installation were 3.9% and 3.3%, respectively [18].

There is growing literature on the factors influencing engagement with health and well-being smartphone apps. In our recent review of 41 studies, we identified 26 factors that are important for the uptake of and engagement with such apps [20]. In addition to a wide range of behavior change techniques (eg, self-monitoring and goal setting) [21,22], several other factors were identified as influential, including the role of health care professionals in the promotion and recommendation of health apps [23] and embedded professional support [24]. The latter was found to be particularly important for certain behaviors (ie, alcohol reduction, suicide prevention, anxiety, and self-harm), with stand-alone apps considered insufficient by users and clinicians [14]. In an assessment of 93 mental health apps, daily minutes of engagement were higher for apps that included peer support (median 35.1, IQR not applicable; n=2) and coping strategies such as mindfulness and meditation (median 21.5, IQR 15) compared with apps that incorporated self-monitoring or psychoeducational features (median range 3.53-8.32) [18]. Few qualitative studies have been undertaken to explore the factors that affect engagement with health and well-being apps. These undertaken studies have focused on specific populations or behaviors. Existing studies have focused on weight loss behaviors and alcohol reduction and have found that the specific content of health information messages [17,22], personalization of app content [25], and the user’s demographics [22] are some of the factors deemed to be important for engagement with weight loss and alcohol reduction apps. Findings from these studies highlight that the specific context in which apps are developed and used will influence user engagement. To date, most studies have investigated the features of health apps that are desirable by a certain population, and little is known about the factors deemed important for engagement with a wider range of health and well-being apps [20]. These studies suggest that the context in which apps are developed and used might often be behavior or population specific. Therefore, this research intends to address this gap by exploring the views on the big 4 public health priority behaviors related to prevention (smoking, alcohol consumption, physical activity, and diet) and mental health. The findings from this study may inform future app development to improve user engagement with apps that target health promotion. The findings may also be particularly useful for stakeholders in public health to inform the development of interventions to promote engagement with evidence-based health and well-being apps, for example, directly contributing to the long-term plan of England’s National Health Service to become *digital first*.

### Theoretical Framework

**Figure 1:**
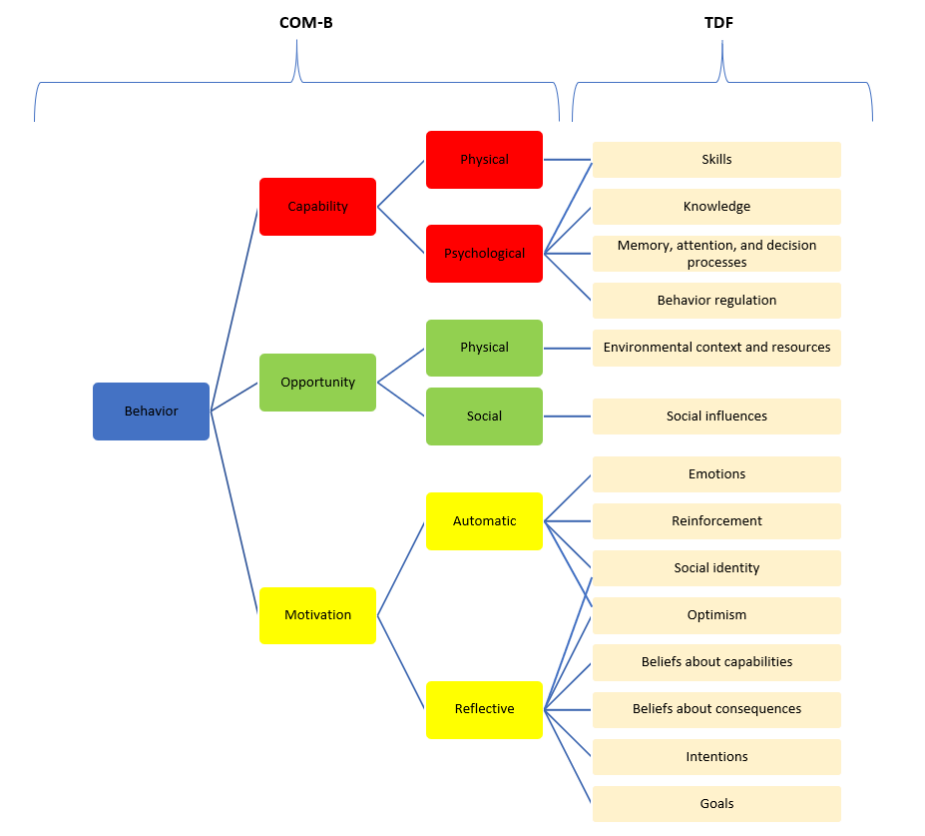
A visual representation of how the Theoretical Domains Framework can be mapped onto the components of the Capability, Opportunity, Motivation–Behaviour model [20]. COM-B: Capability, Opportunity, Motivation–Behaviour; TDF: Theoretical Domains Framework.

The Capability, Opportunity, Motivation–Behaviour (COM-B) model [26] provides a broad framework for understanding the factors influencing user engagement. According to the COM-B model, behavior (eg, app uptake and engagement) arises from the interaction between the individual’s physical (eg, app skills) and psychological (eg, knowledge of using an app) capability, physical (eg, features of the app) and social (eg, recommendations for an app) opportunity, and automatic (eg, feedback received from an app) and reflective (eg, user’s self-confidence in using an app) motivation. The Theoretical Domains Framework (TDF) [27] is a synthesis of 33 theories and 128 psychological constructs and includes 14 domains that can be mapped under the 3 main components of the COM-B model. Taken together, the COM-B and TDF provide a detailed theoretical framework that allows the careful consideration of factors influencing engagement with health and well-being apps (Figure 1 [20]).

### Aim

We aim to investigate people’s experiences and reasons for engaging or not engaging with health and well-being apps using qualitative interviews and map the identified factors onto the COM-B model and the TDF.

## Methods

### Study Design

This qualitative study used semistructured interviews and was designed and reported in line with the COREQ (Consolidated Criteria for Reporting Qualitative Research) checklist (Multimedia Appendix 1) [28]. The study protocol was preregistered on the Open Science Framework [29]. This study was part of a larger project investigating both the uptake of and engagement with health and well-being apps, and the findings on uptake are published elsewhere [30].

Ethical approval was obtained from the Faculty of Medicine and Health Sciences Ethics Committee at the University of East Anglia (reference number 201819–089).

### Participants and Recruitment

Participants were recruited through social media. Recruitment through Facebook is known to be an effective way to reach adults interested in health and well-being apps [31]. It is fast and cost-effective and has been found to provide better representation and improved participant selection compared with traditional recruitment methods [31]. Eligible participants for this study (1) were aged ≥18 years, (2) were able to give consent, (3) owned a smartphone, (4) would consider using a smartphone app to change their behavior in the future, and (5) could travel for an interview. Purposive sampling was used to ensure the diversity of the sample (age, gender, ethnicity, educational level, and employment) [32]. Invitations for interview were sent to 38 participants, of whom 14 (37%) individuals did not respond, and 6 (16%) subsequently canceled for personal reasons, leaving a total of 18 individuals. The recruitment and interviews took place in batches of 3 or 4, and the recruitment was stopped when data saturation was reached.

### Measures

To determine eligibility and describe the sample, data were collected on (1) age; (2) gender; (3) ethnicity, which was measured using the Office for National Statistics’ index; (4) level of education; (5) employment status; (6) whether they had ever used a health or well-being app; (7) whether they currently use a health or well-being app; (8) the last time they downloaded an app; and (9) frequency of app use.

### Procedure

The participants read the information sheet available on the web. Those who expressed interest in participating were required to fill a web-based screening questionnaire to assess their eligibility (Multimedia Appendix 2). The questionnaire was hosted by the *Jisc Online Surveys* (JISC) software. Participants meeting the inclusion criteria received a comprehensive participant information sheet via email and were invited for an interview during which written consent was obtained. Participants received a US $27.50 (UK £20) gift voucher for their participation.

The interviews were conducted by a female researcher (DS) between July 2019 and August 2019. The interviews took place face-to-face in Norwich, England, at the University of East Anglia (17/18, 94%) or at the participant’s home (1/17, 6%). A participant was not included in this study because they had no previous experience of using health or well-being apps. No one else was present during the interviews. The interviews lasted between 26 and 63 minutes. Semistructured interview techniques were used to elicit data on participants’ experiences of and views on engagement with health and well-being apps. The final topic guide was informed by feedback from key stakeholders, including patient and public involvement representatives and domain experts from Public Health England. A think-aloud task (reported elsewhere [30]) was followed by several questions regarding participants’ experiences of engagement with apps (see Multimedia Appendix 3 for the topic guide).

### Data Analysis

The interviews were audio recorded and transcribed verbatim. A framework analysis approach was applied to analyze the data. This followed the stages of familiarization, identification of the thematic framework, indexing, charting, mapping, and interpretation [33]. For pragmatic reasons, a second author (OP) independently coded 3 randomly selected transcripts (representing approximately 15% of the transcripts). The deductive framework was based on the TDF. Through repeated discussions between the first and second authors, the deductive thematic framework was refined iteratively, and discrepancies were resolved through discussions with another author (FN). DS completed indexing using QSR NVivo 12 and charting. During charting, responses were clustered based on the thematic framework. This was followed by mapping and interpretation, during which the data were examined to identify patterns. To increase the trustworthiness of the findings, peer debriefing by the University College London Tobacco and Alcohol Research Group, which has extensive experience in the application of the COM-B model and TDF in health research, was used to ensure the accuracy of data interpretation and data analysis. Peer debriefing is a form of analytical triangulation in which researchers who are not directly involved in the study are prompted to provide input and critical opinions on various aspects of a project [34]. The use of the TDF in the deductive framework analysis approach was particularly useful for coding the results under several factors, which may otherwise have been overlooked. It was expected to explore a large number of factors because the TDF has 14 constructs, as opposed to other well-known methods. However, researchers were aware that the findings would not be coded under all available constructs. The constructs under which no findings were coded were omitted from the *Results* section.

### External Validity

Member checking was conducted to ensure the trustworthiness of the results and further minimize researcher bias [35]. After the interview, participants were told that the researchers might contact them to share the findings and ask their views on the findings. Of the 18 participants, 6 (30%) randomly selected participants were contacted and invited to provide feedback on the summary of the findings and the conclusion. The purpose was to investigate whether participants agreed with the interpretation of the results and whether they felt that their opinions were captured and appropriately presented. Incentives were not offered for their input. Of the 6 participants who were approached, 2 (33%) participants responded and indicated that they agreed with the interpretation.

### Reflexivity

DS, a PhD candidate, conducted the interviews after receiving extensive training in qualitative research methodology and kept field notes and a research journal during data collection. There was no prior relationship established between DS and the participants. The coauthors had experience in mixed methods research and the application of the COM-B model and the TDF. Efforts to establish a good rapport with the participants were made throughout the study. The interviews were individually adapted to the flow of discussion made by each participant. Most participants (15/18, 83%) stated that they wished to find out more about the findings of the research. DS also shared her research interests with the participants after the interview.

## Results

### Participant Characteristics

A total of 18 adults (mean age 43, SD 14 years; range 21-68 years) were recruited, of whom 9 (50%) were female, 14 (78%) were White British, 13 (72%) were employed full time, and 8 (44%) had a college degree or higher. Of the 18 participants, 11 (61%) participants reported currently using at least one health or well-being app at the time of the interview. Of the 18 participants, 3 (17%) participants expressed their intention to change 1 behavior, and all the other participants were interested in changing >1 behavior (eg, losing weight, being more active, and managing their mood). Of the 18 participants, 1 (6%) participant had never used health apps before and did not wish to express their views on engagement; therefore, the findings of this paper are based on the views and experiences of the remaining 17 participants about their engagement with health and well-being apps (Multimedia Appendix 4).

### Factors Influencing Engagement With Health and Well-Being Apps

An overview of the factors mapped under the constructs of the TDF and components of the COM-B can be found in Table 1. All relevant data were coded under 71% (10/14) of the constructs of the TDF. There were no data that could not be coded under any of the constructs of the TDF.

**Table 1:** Perception of factors influencing engagement with health apps.

COM-B^a^ model component, TDF^b^ construct, and factors				Description
**Psychological capability**				
	**Knowledge**			
		User guidance	Instructions on how to effectively use a health app	
		Statistical information	A visual or numerical summary of progress or quantification of the behavior	
		Health information	Educational information related to health and well-being aspects	
	**Memory, attention, and decision processes**			
		Reduced cognitive load	The app is not too time-consuming, is easy to use, and requires minimal input	
		Reminders	Preferably customizable notification-type messages	
	**Behavior regulation**			
		Self-monitoring	The ability of the app to support self-regulation of the target behavior	
		Routines	The ability to support routine or habit formation	
		Safety netting	Retaining the app for a potential upcoming event in the future	
		*Stepping-stone*	App as a first step in the behavior change process	
**Physical opportunity**				
	**Environmental resources**			
		Tailoring	Innovative features, adaptability, and interactive and 2-way communication between the app and user	
**Social opportunity**				
	**Social influences**			
		Peer support	Including social interaction with users with similar needs within the app or within their community; a choice to connect to social media platforms, competitions, and challenges with others or with themselves	
		Social support (practical)	Possibility to contact health professionals and practitioners within the app	
**Reflective motivation**				
	**Beliefs about capabilities**			
		Self-confidence	Perceived capability to change one’s behavior using an app	
	**Goals**			
		Goal setting	Establishing what the user would like to achieve	
		Action planning	Establishing how the user would like to achieve set goals	
	**Beliefs about consequences**			
		Commitment	The level of commitment while engaging with an app to change the behavior and achieve set goals	
**Automatic motivation**				
	**Reinforcement**			
		Feedback	Feedback regarding the user’s performance	
		Rewards	Tangible (eg, objects and discounts) and intangible (eg, badges and certificates) rewards in response to the user’s effort and gamification elements	
		Encouragement	Additional ways to provide reinforcement (eg, encouraging messages)	
	**Emotions**			
		Positive emotions	Triggered by the included user guidance, statistical information, additional health information, embedded professional support, community networking possibilities, tracking features, and rewards	
		Negative emotions	Triggered by lack of user guidance, invasive push notifications, cognitive overload, and unrevealed in-app costs	
		Mixed emotions	Triggered by reminders	

^a^COM-B: Capability, Opportunity, Motivation–Behaviour.

^b^TDF: Theoretical Domains Framework.

#### Capability to Engage With Health and Well-Being Apps

##### Knowledge

Many participants perceived their knowledge on how to use an app and the embedded statistical and health information as an important influence on their engagement with an app. We inferred this from the desire that many people reported for clear *user guidance* and, in some cases, for help on how to increase their capability to perform a behavior (eg, demonstration of the behavior). A participant explained that they had stopped using an app in the past owing to “insufficient guidance on how to use it” (Participant 8):

So this is where I start getting, well why are you asking me these questions if you’re not going to let me carry on with it and that’s where I start getting confused, going back, not really understanding where I need to go from here.

Participant 15

Furthermore, the importance of *statistical information* about their progress and achievements was reported by most participants:

It’s nice to see your progress on a graph and it’s just very clear. It’s a single screen, you have icons for all the activities that you’ve done during the day.

Participant 6

In addition, most participants expressed the need for relevant and comprehensive *health information*:

Knowledge is key.

Participant 14

Several participants stated that having embedded educational articles would help them to build knowledge and understand and manage their behavior better. Not getting enough health information was reported as the main reason for a participant to look for a different app:

It’s got to have the information that I want and have it easily accessible.

Participant 2

##### Memory, Attention, and Decision Processes

Participants perceived *reduced cognitive load* and customizable notification-type *reminders* as factors that positively affect their capability to engage with an app. All participants described favoring apps with *reduced cognitive load*. This included apps with limited complexity, less data input, and a limited number of available features to choose from.

A participant suggested that an app should apply a multilevel approach with “a light version of an app and then enhanced” (Participant 15). They described that an app might have a simple version for basic users with no registration and minimum data input and a more advanced version with all features available for power users.

Several participants expressed that a time-consuming app would be immediately deleted:

A mood tracker is something I probably wouldn’t use because it looks like it would require a lot of data of me putting in and typing it on to stuff.

Participant 7

Although push notifications were considered more or less annoying, many participants described *reminders* as being particularly useful. A participant described that not being reminded to engage with an app led him to disengage:

Because I wasn’t reminded, I stopped using it. And I think that’s really important.

Participant 1

However, a few participants who reported not finding notifications useful stated that they would immediately turn the reminders off or delete the app:

I’m sure there are many apps I’ve deleted because of reminders.

Participant 7

Others have suggested that *reminders* might cause harm. For example, a participant described uninstalling a smoking cessation app as the reminders periodically reminded them about their addiction, thus serving as a prompt that induced cigarette cravings. However, 12% (2/17) of participants proposed that opting in to receive reminders would be desirable instead of opting out. In addition, a participant suggested that human-like reminders in the form of SMS text messages would be less likely ignored and would create the perception of a human touch within the app:

I think text messages would work better because I don’t ignore my text messages and my WhatsApp messages because there’s real people connected to those, you know? ... if I could think of an ideal it would be a text message that kind of asked you a question and you replied and it felt like it was a human being.

Participant 6

##### Behavior Regulation

Participants perceived that *self-monitoring*, established *routines*, and *safety netting* and *stepping-stone* characteristics of the app would enhance their engagement with the app.

All described *self-monitoring* features as important in behavior regulation, even when there is no particular goal set or when achieving the goal shows a delay:

Monitoring, really because the goal is probably going to go a bit by the wayside because work has been too busy and life has changed and lots of stuff has happened this year. So I’m behind my goal but I still use it as a monitor.

Participant 17

Some participants reported that a daily *routine* of using an app would make engagement with it more accessible and continuous. Of the 17 participants, 2 (12%) participants described how using a weight management app for a week was necessary for them to get into a routine and helped them stay engaged after that. However, one of them explained that it was difficult to use the app in the beginning, although after a few days, it got easier.

A number of participants explained that they perceived physical activity apps as *stepping-stones* to physical activity services, with the app acting as an intermediate tool in behavior change. Of the 17 participants, 2 (12%) participants described that an app helped them to get enough experience and practice home workouts that boosted their confidence to eventually sign up for a gym membership:

You can just literally do it at home [fitness app] until you feel I suppose a bit more confident to go out and join [the gym].

Participant 10

Many of the participants described apps as a *safety netting* tool (eg, relapse prevention). Several participants reported a tendency to reengage with a weight management app periodically and when necessary to regulate their weight, for example, before or after a holiday season or an important upcoming event because the app had helped them achieve their goals in the past:

I think I have periodically come back to it and thought “no it worked before, it’ll work again.”

Participant 13

#### Opportunity to Engage With Health and Well-Being Apps

##### Environmental Resources

Participants perceived that *tailoring* the technology was a factor that would influence sustained engagement. Many participants expressed the need for features that would create a better physical opportunity to engage with an app and a more personalized experience during the engagement. Many participants described seeking to engage with apps that provide 2-way communication, which can adapt to the person’s needs based on how they interact with such tools. Several participants mentioned the inclusion of innovative features. These features comprised embedded artificial intelligence (AI) to receive health-related advice and tailored content; facial recognition; and recognition of nonverbal cues for better outcomes in physical activity, for example, correcting posture; and using the phone’s camera to provide nutritional data of cooked food:

If it’s smart, as well. Has it got a little bit of artificial intelligence built into the background? Is it using my data? Is it saying “do you know what? Actually, you’ve done really well this week, you’ve used the app this amount of times. How are you feeling?”

Participant 2

A participant described that the lack of novelty of an app would lead them to disengage with it. In contrast, another reported the opposite—they would feel put off if they would need to learn new features:

It’s no good downloading an app and then six months later looking at that app and it’s still the same, that would stop me.

Participant 14

If something’s working we want it to stay as it is, we don’t want it to change, and even if there are improvements to it, if it’s new it can kind of put people off in a way.

Participant 13

Syncing with wearables or other additional devices was described as desirable by many.

##### Social Influences

Peer support and social support (practical) were perceived by participants as factors that may sustain engagement with an app. Several participants perceived networking within a web-based community as necessary peer support. Some described that sharing and exchanging experiences with others would encourage and motivate them in their journey. Others suggested anonymity for users and moderation of discussions to avoid “misinformation” (Participant 12):

I like the idea that it’s round the clock support, because so very often with mental health issues it’s kind of 2 o’clock in the morning that they are the worst, and that is when you need to talk to somebody, and the idea of having a community who you don’t have to explain how you’re feeling sounds really good.

Participant 11

Embedded social media to share their progress with others was reported as a useful feature only by a few participants who were using physical activity or weight management apps. However, a couple of participants highlighted that this feature should be optional. Physical activity and weight management app users also described challenges and competitions as motivating and fun:

There’s challenges, which will help you with your weight loss, your fruit and vegetable intake, the exercise challenges that you can do, either with yourself or your friends, which are good for motivation.

Participant 15

All participants expressed their preference for an app that would offer built-in professional support, such as health practitioners, coaches, and dieticians (for *social support* and *practical support*). A participant with an existing medical condition described the need for health practitioner support within an app. In addition, 12% (2/17) of participants described that built-in support would help with accountability, and 6% (1/17) of participants indicated that they would be willing to pay to access an app with in-built support. Another participant commented that the embedded professional support was the best feature of a mental health app they were using:

Yeah if you could sort of talk to a healthcare professional in that app I think that would be better, because then they would have the up to date I suppose treatments and methods so that you know you’re not going on old information.

Participant 10

I: If you would need to say just one thing that is the best in the app, what would that be? P: The support.

Participant 11

#### Motivation to Engage With Health and Well-Being Apps

##### Beliefs About Capabilities

Apps were perceived by several participants as useful tools to enhance their *self-confidence* in changing their behavior. A participant described that the community networking opportunities further helped her *self-confidence* and motivated her to use the app:

The app made me feel more confident in doing it, even it was just basic home exercises.

Participant 7

##### Goals

*Goal setting* and *action planning* were perceived as key factors for sustained engagement and motivators of behavior change. *Goal setting* was reported as being valuable by all participants in addressing behavior change; however, half of the participants described the need for *action planning* features to help them achieve their set goals:

I’d want something which was a bit more than press one button every day to say you haven’t smoked; it was great for the first 10 minutes of using the app because I got all this information about “wow thousands of pounds and the health benefits,” and then after that it was literally just press this button to say you haven’t smoked, and that wasn’t really enough for me.

Participant 13

##### Beliefs About Consequences

Several participants expressed that their level of *commitmen*t to achieve their goal shaped the level of engagement with the app they used:

The app, the initial—the main reason you’re on that app is to get your result of what you want to achieve, what you want to do to help you stay on track.

Participant 9

##### Reinforcement

Many participants perceived *feedback*, *rewards,* and *encouragement* as automatic motivational factors that may sustain engagement with an app. A number of participants expressed that they needed continuous *feedback* to reinforce their continuous use:

I think an app that might give you feedback, a notification, that would keep me entertained and would keep my level of focus and wanting to continue with it.

Participant 3

Intangible *rewards* (ie, badges and certificates) were described as another form of reinforcement by several participants for motivating them and as *“nice”* (Participant 14) or something to *“show off”* (Participant 5). However, some other participants described intangible rewards as *irrelevant*. They reported that the tangible rewards they received in the past, including cinema tickets, lower insurance premiums, and loyalty points that can be exchanged for objects or a free water bottle, provided better motivation to engage with the app than intangible ones. In addition, a few participants expressed the need for *encouragement* in the form of motivational messages:

In this context, so badges, you earn nine of 24 badges so far. For me a little bit irrelevant actually, what are you going to do with it, there’s other reasons why you’re quitting, not to get the badges.

Participant 16

##### Emotions

Participants expressed positive emotions regarding available user guidance, statistical information, additional health information, embedded professional support, the possibility for community networking, self-monitoring features, and rewards. However, negative emotions were expressed for the lack of user guidance, invasive push notifications, and cognitive overload. Finally, feedback on reminders was person-dependent and triggered mixed feelings across participants.

## Discussion

### Principal Findings

This study applied the COM-B and the TDF to explore users’ views about factors that influence engagement with health and well-being apps. We found that *knowledge*, such as user guidance and statistical information; *memory, attention, and decision processes*, such as reduced cognitive load; *environmental resources*, expressed by the tailored technology; and *social influences,* referred to as peer and professional support, are the most important factors for participants’ engagement.

Many factors identified in this study are consistent with those in previous literature. Previous research has found that engagement with health apps is greatly influenced by factors affecting users’ capabilities, including different types of knowledge (user guidance, statistical information, and health information) [20,36], reduced cognitive load, reminders, and self-monitoring features [20,22,37]. These factors could be targeted during app development updates of existing apps to improve user engagement. In line with previous findings, reminders were not found to be universally useful [20]. A possible explanation is that reminders may be behavior-dependent and person-dependent. Some participants reported that they had stopped engaging with a health app because they were not reminded to continue using it, whereas others tended to ignore or delete the apps that sent reminders. This research is the first to identify a novel factor, the perception of certain apps as *stepping-stones* to more intensive behavior change. For example, a home-based workout app or a walking app could seek to provide enough self-efficacy and competence for an individual to join a gym or start using a running app. An explicit *stepping-stone* approach could be a useful addition for apps targeting behaviors that are harder to achieve because of negative emotions, such as embarrassment, shame, or pressure, including those targeting sedentary behavior. This novel finding shows that sustained engagement is not always necessary to support desired health and well-being outcomes through additional behavior change activities.

Engagement is further influenced by the users’ physical opportunities, such as tailored technology, and social opportunities and peer support, including community networking, embedded social media and social competitions, and professional support [20,24,25,37]. Some users would want the app to be based on machine learning opportunities and on 2-way interaction with users. The adaptable nature of an app and the provision and level of AI included may also play a key role in engagement. These factors may be harder to include once an app is developed; therefore, it might be important to consider these aspects during the development process. Indeed, such tailored technology may be the most important aspect to consider. For example, although there may be financial considerations precluding the provision of personal, professional support within an app, this service may be developed using AI. These forms of technological personalized models in health behaviors such as nutrition or smoking, including machine learning models, have been suggested to aid the process of making decisions about diet and food [38]. However, AI has not yet been found in diet monitoring apps [39]. A randomized controlled trial found that participants allocated to an advanced version of a smoking cessation app with an AI chatbot had 107% higher engagement with the app and over twice the odds of being abstinent at 1-month follow-up compared with participants using the standard version of the app [40]. Furthermore, timely AI-based behavior change support received just in time may further increase behavior change. Although unguided interventions can be effective, having professional support within an app tends to increase effective engagement [16]. Simple interventions that do not require professional support can be more widely disseminated and are more cost-effective than those with embedded professional support [16].

Users’ reflective motivation, including beliefs in their capabilities (self-confidence), consequences (commitment), and goals (goal setting and action planning), is essential for engagement. Although the first 2 factors are harder to address because they are within-person factors, the latter can be easily implemented as features of the app. A possible way to increase self-confidence and commitment is perhaps to address these within the app by using quizzes or articles [41], (eg, for commitment, “How to stay on track to achieve your goal?”) or check-in messages using AI [40].

Emotions are considered as automatic motivation factors and are a powerful driver of behaviors that affect adherence, for example, engagement with a health app [42]. It is noteworthy that we did not identify emotions directly influencing engagement, or we failed to identify them. However, we found evidence that the other factors affected participants’ emotions. Appealing features, such as statistical and health information, embedded peer and professional support, and tracking features and rewards, triggered positive emotions. In contrast, a lack of user guidance, invasive notifications, and cognitive load triggered negative emotions. A better understanding of how the presence or absence of specific features affect participants’ emotions may be useful for the development of new apps or the refinement of existing apps, which, consequently, may lead to better engagement with health apps.

### Strengths and Limitations

A strength of this study is the methodology used. First, to assure that the research was as relevant and meaningful as possible, the study protocol was developed with policy maker and patient and public involvement representatives in the design of the topic guide. Second, the research was further informed by well-established theoretical models; the COM-B and the TDF and peer debriefing were used to help data interpretation and data analysis [34]. Third, the purposive sampling technique allowed the recruitment of a diverse sample regarding gender, educational level, and employment status. Finally, member checking was conducted, which is a technique used to establish the credibility of the findings by sending a brief summary of the findings to randomly selected participants [35].

This study has several limitations. The recruitment of a sample of participants with more diverse demographics might have identified additional factors that are important for engagement. Several participants were not using health or well-being apps at the time of the interviews and had not downloaded any health and well-being apps during the 6 months before the interview. This may have led to limitations associated with the challenges of retrospective recall. Although the research aimed to recruit a heterogenous sample to capture a wide with *big 4* public health priority behaviors related to prevention (smoking, alcohol consumption, physical activity, and diet) and mental health apps, a homogenous sample may have allowed for a more in-depth understanding of engagement with apps for specific behaviors. The study only included participants who considered using a smartphone app to change their behavior in the future. Including participants who have used health and well-being apps in the past but are less receptive to using them now may have provided additional perspectives on factors influencing app engagement. The findings may be influenced by the intention-behavior gap, with participants reporting on factors perceived as important for changing their behavior through an app. However, this does not mean that they would act on their intention. An example of this is the finding that many participants wanted access to a web-based community. Although web-based communities typically suffer from the *90-9-1 principle*, whereby the content in web-based communities is generated by 1% of the members, with 9% editing or modifying it and 90% being passive observers [43], this may not be the case with a closed community built to support behavior change, where individuals are seeking support from each other. In addition, the meaning of the term *engagement* was not explicitly defined during the interview when individuals shared their experiences and views of engagement. Their interpretation of engagement is likely a mixture of microengagement and macroengagement, and a distinction between these 2 levels of engagement was not considered when interpreting the findings. Finally, of the 6 participants who were contacted, only 2 (33%) responded to the request for external validation. Therefore, it is not clear whether the other participants disregarded our request or disagreed with our interpretation.

### Implications and Future Research

This research provides insight for stakeholders in public health, policy makers, and developers of apps that target disease prevention and health promotion. Our findings may also be used to inform the development of interventions aiming to promote engagement with evidence-based health and well-being apps. In the United Kingdom, this aligns with the priorities of the National Health Service’s long-term plan (ie, *digital first*).

Our main finding is centered around providing the necessary support for increased engagement with health apps. We found that embedded professional support may have a substantial impact on engagement, although it may not be beneficial for all health behaviors. Embedded social support may be particularly important for some behaviors that are more likely to be complex and require intensive support to maintain engagement. These behaviors are the ones that require reassurance, guidance, or emotional support [16], such as apps targeting substance misuse or those developed to improve mental health. Although it is not always feasible to develop an app with embedded professional support, there might be different ways outside of the app to address this need. For instance, there may be a way to provide support within community-based care to assist with the uptake of health apps and with the progress of or potential barriers to engagement. Another way to mitigate the absence of embedded professional support is to investigate the potential efficacy of advanced computational techniques, such as AI, to mimic the support provided by health care professionals (eg, in the form of chatbots or other types of conversational agents). There is an urgent need for more research on the optimal type (eg, technology-mediated or *blended*) and timing of support needed within various health and well-being smartphone apps.

To better meet users’ needs, the design of apps would ideally be informed by a user-centered and iterative development process, supported by mixed methods research, including in-depth interviews. As app engagement is generally greater in those with higher socioeconomic status [44], involving individuals with lower socioeconomic status is particularly important [16]. Furthermore, people who are directly affected by the digital divide or digital exclusion and who may struggle to benefit from health apps due to lack of skills or low digital literacy could be targeted by offering app-use tutoring. Although this may require investment or relocation of resources within community health care settings, it may increase the reach of health apps and lead to a greater public health benefit. Furthermore, we noted a possible tension between users wanting the app to be easy to use (which may be facilitated by providing user guidance) but at the same time not too time-consuming. As the provision of user guidance helps individuals with limited technological skills, we believe that such features should still be prioritized. Undoubtedly, finding the balance between producing an app with all features necessary for behavior change to occur and ensuring that the app is intuitive enough will pose a challenge for app developers.

In addition, more experimental research would help us to better understand the effects and potential interactions among the engagement factors identified in this study, including usability (ease of use), reminders, embedded support, rewards, and goal management. Textbox 1 provides a summary of considerations to help app developers and commissioners design interventions to increase effective engagement. These factors are structured around COM-B and TDF.

**Considerations for policy makers, industry, health care providers, and app developers for maximizing engagement with health and well-being smartphone apps.**

**Capability, Opportunity, Motivation–Behaviour model components and considerations for policy makers, health app portal providers, and app developers**

**Capability**

- Provide user guidance on how to use an app, visual or numerical summary of progress, and evidence-based additional health information related to the behavior targeted by the app
- Minimize the time required to use the app where possible
- Provide customizable reminders that users could opt out from
- Provide the option to self-monitor features
- Promote safety netting and relapse prevention features such as the possibility to restart or reengage with the app later
- Promote a routine for engagement with an app, for example, highlighting the role that routine may play in the effectiveness of an app

**Opportunity**

- Collaborate with interaction design experts and end users to enhance the aesthetics of apps
- Provide the possibility for community networking within the app and linking to social media as an optional feature to share progress where appropriate
- Offer the possibility for social competition and challenges where appropriate
- Consider the provision of embedded professional support and, if this is not feasible, providing offline one-to-one support with the uptake of and the engagement with health apps; this may improve motivational factors, such as commitment, self-confidence, and perceived competence of engaging with a health app
- We advise that exploration be made for where engagement enhancement could be made with appropriate and proportionate machine learning, artificial intelligence, or other forms of learning systems.

**Motivation**

- Develop a time-efficient app that would require as much engagement as is required to achieve the desired outcome; this might be different for different behaviors
- Include reinforcement in the form of feedback, encouraging messages, and rewards
- Offer intangible rewards, such as certificates or badges
- Offer tangible rewards that can be converted to discount in other places (eg, health insurance providers, pharmacies, or sports parks)
- Include goal setting and action planning features on how to achieve set goals (when applicable)
- Take into account the user’s emotions about certain features by involving users in the development and update of health apps as the lack of some features could provoke strong negative emotions such as disappointment and might lead to rapid disengagement

### Conclusions

People perceive their capability to engage with an app as an important influence on their sustained engagement with it. This perception was inferred from people’s desire for apps to contain clear user guidance, require less cognitive load, and support easy self-monitoring. Tailored technology and peer and professional support may influence users’ opportunity to engage with an app, and goal setting with action planning may play a key role in the motivation to engage with an app.

## Appendix

Multimedia Appendix 1 COREQ (Consolidated Criteria for Reporting Qualitative Research) checklist.

Multimedia Appendix 2 Screening questionnaire.

Multimedia Appendix 3 Topic guide.

Multimedia Appendix 4 Characteristics of the included participants.

## Abbreviations

AI: artificial intelligence
COM-B: Capability, Opportunity, Motivation–Behaviour
PHE: Public Health England
TDF: Theoretical Domains Framework
