# Supplementary material for: Perceptions of Factors Influencing Engagement With Health and Well-being Apps in the United Kingdom: Qualitative Interview Study
Source: JMIR Mhealth Uhealth. 2021 Dec 16;9(12):e29098. doi: 10.2196/29098 (PMC8726027; doi:10.2196/29098)
Supplement: Multimedia Appendix 3 [file mhealth_v9i12e29098_app3.pdf]

Multimedia Appendix 3.

Topic guide

Think aloud exercise:

*'In the questionnaire you have mentioned that you would like to [**change a behaviour**]. Imagine that you are now looking for an app for that. Imagine that you are at home and have decided to use an app for that. Please look for an app. You can use your own phone or this laptop if you wish. **[Waiting to see where the participant would look for the app. Use of prompts to think-aloud.]**'*

'I would like to show you a different app pool on this laptop. Please repeat the first exercise but this time use this portal to find an app.'

'You have mentioned that […]. Can you elaborate on that? '

*'How did it feel to search for an app on this portal, instead of [**where they have searched for the first time**]? Why?'*

Follow up questions:

'You have mentioned that [**…**]. Can you elaborate on that?'

'In your view, is there anything missing from this portal? '

Further questions:

'How do you think other people select an app?'

'Why would anyone choose to use an app to change their behaviour?'

'You have mentioned in the questionnaire that you have used/are using [***name of the app***]. How did you find that app?'

'Why have you used it?' **OR** 'What makes you to continue using it?'

OR 'Why have you stopped using it?' **AND** 'Is there anything that would have made you continue to have used it?'

'If it would be your decision, what would you do to promote the use of health apps?'

Additional/final question

Is there anything else you wish to add or anything we haven't covered, and you feel it would important to share?
